# New ecological method for determination of different β-lactams: application to real human plasma samples

**DOI:** 10.1039/c9ra02671a

**Published:** 2019-06-27

**Authors:** Nehal F. Farid, Nada S. Abdelwahab

**Affiliations:** Pharmaceutical Analytical Chemistry, Faculty of Pharmacy, Beni-Suef University Beni-Suef Egypt nadasayed2003@yahoo.com nehalfayek@gmail.com +20 1285999726 +20 1277950994; Pharmaceutical Chemistry, Faculty of Pharmacy, Nahda University (NUB) Beni-Suef Egypt

## Abstract

Recently, the use of antibiotics has become widespread all over the world resulting in bacterial resistance to these antibiotics, which requires alternative medications or higher doses of antibiotics. Implementation of an easy analytical method that can analyze a wide range of β-lactam antibiotics in a single run is important to reduce the time of therapeutic drug monitoring (TDM) in hospitals and minimize the spreading of bacterial resistance. A novel environmentally harmless HPTLC method was developed and validated following FDA recommendations for analysis of four β-lactams; cefaclor, cefotaxime, cefepime, and meropenem, in human plasma. A solvent mixture of ethylacetate : methanol : deionized water : formic acid (60 : 30 : 15 : 1, by volume) was the used developing system, detection was carried out at 270 nm, and valacyclovir was used as an internal standard. A lower limit of quantitation (LLOQ) was found to be 0.1 μg per band for all the analyzed drugs. Validation parameters were calculated and found to fulfil the international requirements for bio-analytical method validation. Additionally, each of the studied antibiotics was given to a group of healthy volunteers from which blood samples were collected at *t*_max_ of each, methanol was used for precipitation of plasma protein, and the developed method was used for calculation of the concentrations in the separated plasma samples. The developed method, being a green one, and time and money saving, can be used for TDM of these drugs in clinical studies as well as for quality control analysis in pharmaceutical companies. The proposed method is the first developed HPTLC method for the simultaneous bio-analysis of the selected β-lactams.

## Abstract 2

An innovative ecofriendly HPTLC method was established for *in vivo* analysis of four β-lactam antibiotics with minimal sample pretreatment.

## Graphical abstract

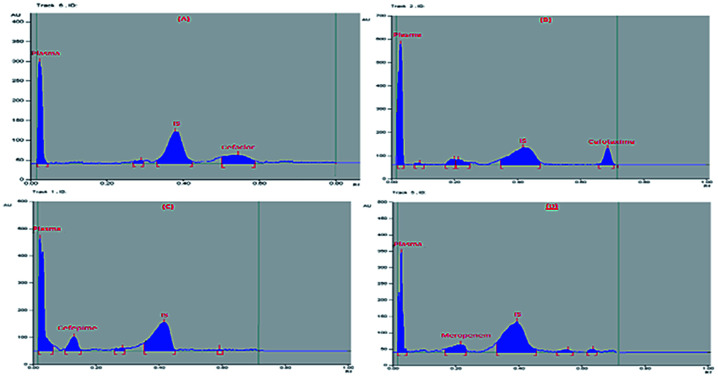

## Introduction

β-Lactam antibiotics are the most commercially used antibiotics in the world, they work by inhibiting cell wall biosynthesis in bacteria.^1^ Cephalosporins and carbapenems are two classes of β-lactam antibiotics that are prescribed for serious infections that involve a wide bacterial range. The drugs under study include cephalosporins [cefaclor (2^nd^ generation), cefotaxime (3^rd^ generation) and cefepime (4^th^ generation)], and meropenem which is an example of carbapenems.

Cephalosporins are broad-spectrum antibacterial drugs; they are the second largest class of β-lactam antibiotics with excellent safety profiles. Third and fourth generations are able to penetrate the blood–brain barrier and reach the central nervous system with sufficient concentrations; thus they are effective in the treatment of meningitis. On the other hand, carbapenems have the broadest spectrum activity and greatest potency against Gram negative and Gram positive bacteria, hence they are used as last resort antibiotics for resistant bacteria,^2^ so they are considered lifesaving drugs.^3^

Appraising the literature, different methods were published for analysis of β-lactam antibiotics in different biological fluids (cefotaxime and cefepime reviews were reported by Consortti and Salgado^4^ and Omkulthom^5^). The published methods for β-lactam antibiotics include HPLC,^6–15^ LC-MS-MS,^16–23^ and capillary electrophoresis.^24–28^ Few TLC densitometric methods were found for analysis of different pharmaceutical formulations containing β-lactam antibiotics.^29–34^ Only one TLC densitometric method was published for *in vitro* determination of cefepime.^35^ Based on the literature survey, no HPTLC method was found in the literature for the *in vivo* determination of the studied β-lactam antibiotics.

Planar chromatography has wide applications ranging from simple screening tests to complicated instrumental quantitative analysis of different samples in different matrices. TLC is one of planar chromatographic methods which has the advantages of time and money saving.^36,37^ It has extensive applications in pharmaceutical analysis,^38,39^ identification of impurities,^40,41^ isolation and separation of biomedical metabolites or constituents from different body fluids with minimum sample pretreatment.^42,43^ It is also used for separation of identical compounds in a mixture.^36,37^ Recently, HPTLC is an improved form of TLC with better resolution and more accurate quantitative measurements. Complex mixtures can be visualized on HPTLC chromatograms at a glance.^37^

One of the significant problems all over the world is the environmental pollution due to the wide use of hazardous chemicals and solvents. The concept of green chemistry developed as natural evolution of pollution prevention. From the goals of green chemistry is to use alternatives to hazardous substances and to develop new analytical methods that can reduce waste and toxic solvents.^44^

Paying attention to environmental issues and the importance of β-lactam antibiotics, the novelty of this work is to develop and validate an ecological HPTLC method to monitor the studied β-lactam antibiotics in human plasma and to ensure a safe and effective treatment for patients, hence, decrease the extensive use of these antibiotics and minimize their bacterial resistance. Furthermore, the method was validated according to FDA guidelines^45^ and all results agreed with the acceptance limits. The most striking features of the proposed method are its simplicity and short analysis time. Furthermore, it is the first HPTLC method for quantitation of the cited drugs in human plasma with minimal sample pretreatment.

## Materials and methods

### Instruments

#### For preparation of plasma samples

Rongtai variable volume micropipette (0.1–100 μL) (Mainland, Shanghai, China) was used for taking samples accurately, while for mixing samples 250 VM vortex mixer (Hwashin, Seoul, Korea) was used. Centrifugation and separation of the precipitated plasma protein was carried out by using 80-2C Low-speed Electric Centrifuge (4000 rpm) (12 tube × 20 mL) (Zjmzym, China) was used.

#### For HPTLC method

The used stationary phase was HPTLC aluminum plates (20 × 15 cm) pre-coated with silica gel 60 F_254_ with 200 μm thickness and 5 μm particle size (Merck, Darmstadt, Germany). Samples was applied as bands using a Linomat V applicator with 100 μL syringe. Scanning was done by TLC scanner, model 3 S/N (Camag, Muttenz, Switzerland) controlled with winCATS software (version 3.15).

Optimization of the method was carried out using a short wavelength, 254 nm UV lamp (Vilber Lourmat, Marne La Vallee, Cedex, France).

### Standards and reagents

- Cefaclor was provided by Pharco (Alexandria, Egypt), Alex for Chemical Industries & Drugs (Alexandria, Egypt) and certified to have a purity of 98.98%.

- Cefotaxime sodium was supplied from Egyptian International Pharmaceutical Industries Co. (E.I.P.I.Co.), 10^th^ of Ramadan City, Industrial Area, Egypt, with a purity of 99.02% according to the manufacturer certificate of analysis.

- Cefepime hydrochloride, with a purity of 98.95%, was given as a gift by Pharco B International, New Borg El Arab City, Third Industrial Zone, Alexandria, Egypt.

- Meropenem was purchased from (Sigma-Aldrich Chemie GmbH, Germany), with a purity of 98.89% purity.

- Valacyclovir with purity of 99.21% according to the supplier certificate of analysis and was provided by Hikma Pharma (6^th^ of October City, Egypt).

- Ethylacetate, methanol, formic acid (EL-Nasr pharmaceutical, Chemical Co., Abu Zabaal, Cairo, Egypt).

- Deionized water (SEDICO Pharmaceuticals Co., 6^th^ October City, Egypt).

### Pharmaceutical formulations

- Bacticlor® (0.5 g cefaclor per capsule), manufactured by Pharco (Alexandria, Egypt), Alex for Chemical Industries & Drugs (Alexandria, Egypt).

- Cefotax® (1 g for I.M. or I.V. injection), is labeled to contain 1048 mg cefotaxime sodium (equivalent to 1000 mg cefotaxime), and is manufactured by Egyptian International Pharmaceutical Industries Co. (E.I.P.I.Co.), 10th of Ramadan City, Industrial Area, Egypt.

- Forcetex® (1 g for I.M. or I.V. injection), containing 1213 mg cefepime hydrochloride (corresponding to 1000 mg cefepime) and is manufactured by Pharco B International, New Borg El Arab City, Third Industrial Zone, Alexandria, Egypt for Novartis Pharma. Cairo, Egypt.

- Meronem® (0.5 g for I.V. injection) is manufactured by ACS Dobfar SpA, Italy for AstraZeneca UK Limited, Macclesfield, Cheshire, SK 10 2 NA, United Kingdom.

### Blank plasma samples

- Blank plasma samples were provided by Dr Khaled Nagy Laboratory, Beni-Suef, Egypt and they were collected from healthy voluntary donors to be used as a blank matrix.

### Chromatographic conditions

The used stationary phase was HPTLC aluminum plates (20 × 15 cm) pre-coated with silica gel 60 F_254_ with 200 μm thickness and 5 μm particle size (Merck, Darmstadt, Germany) and the mobile phase was consisted of a solvent mixture of ethylacetate : methanol : deionized water : formic acid (60 : 30 : 15 : 1, by volume). Samples was applied using Camag Linomat V applicator as bands of 4 mm width, 5 mm apart from each other, and 15 mm from the bottom edge of the plate. Chromatographic development was done in a glass jar saturated with the mobile phase mixture for 15 min. The temperature was maintained constant at 25 °C and UV scanning was carried out at 270 nm for all the studied drugs.

### Solutions

Cefaclor, cefotaxime, cefepime, meropenem, and valacyclovir (IS) stock solutions (1 mg mL^−1^) were separately prepared in methanol using five separate 25 mL measuring flasks.

### Calibration curves

#### For pure standards

Calibration curves were constructed in the concentration range of 0.06–3 μg per band for the studied antibiotics. Different pure samples of each antibiotic in the range of 60–300 μg mL^−1^ were separately prepared in a set of 10 mL calibrated flasks using the corresponding stock solution (1 mg mL^−1^). 1 mL of valacyclovir (IS) stock solution (1 mg mL^−1^) was accurately added to each sample and the volume was completed with methanol. 10 μL of each sample was applied in duplicates to HPTLC plates and the steps mentioned under chromatographic conditions were then followed. The integrated peak area ratio (integrated peak area of the analyte/peak area of IS) was calculated and used to construct the calibration curves and computing the corresponding regression equations.

#### *In vitro* calibration standards and quality control (QC) samples

*In vitro* calibration curves were constructed in the range of 0.1–3 μg per band. Various concentrations each of the studied components in the range of 100–300 μg mL^−1^ were prepared from their particular stock solutions (1 mg mL^−1^) into four sets of 10 mL volumetric flasks. One mL each of drug free plasma and IS stock solution (1 mg mL^−1^) were separately added to each sample, and then the volume was adjusted with methanol. The prepared samples were vortexed for one min and the precipitated plasma protein was then removed by centrifugation at 4000 rpm for 5 min. 10 μL of the clear supernatant of each sample was then applied in duplicates to HPTLC plates following the instructions previously given under chromatographic conditions. Quality control samples were prepared in the same manner as calibration standards with concentrations of 0.6 μg per band (LQC), 1.5 μg per band (MQC), and 2.5 μg per band (HQC). Calibration standard samples were freshly prepared at the time of analysis while QC samples were kept at −20 °C during method validation period.

### Administration of the studied drugs and collection of plasma samples

Blood samples were collected from healthy volunteers (informed on the experimental procedures, the nature of the study, and gave a written approval). Volunteers were divided into four groups (*n* = 6), group I, received a dose of 0.5 g Bacticlor® capsules, group II, took 1 g I.V. dose of Cefotax® injection, group III was injected I.V. with 1 g Forcetex® injection, and finally, group IV was injected I. V. with 0.5 g Meronem® injection. The age of the volunteers ranged from 20 to 45 years old with a weight range 50–75 kg. Blood samples of 5 mL were collected at the specified time of each drug (*t*_max_) (*t*_max_ of cefaclor = 50 min., cefotaxime = 2–5 min., cefepime = 30 min., and meropenem = 5–6 min.^46–49^) in heparinized tubes and centrifuged at 4000 rpm immediately after receipt. The separated plasma samples were stored at −20 °C till the time of analysis.

### Preparation of the collected plasma samples

Previously collected plasma samples were thawed to room temperature just before the extraction and the preparation procedures.

#### For cefaclor collected samples

In 5 mL centrifuge tubes, 1 mL of each sample was accurately transferred, then 40 μL of IS stock solution (1 mg mL^−1^) was added, and the volume was adjusted to 2 mL with methanol. Samples were mixed well for one min and then centrifuged for 5 min at 4000 rpm. 50 μL of the clear supernatant was then applied in triplicates to HPTLC plates.

#### For cefotaxime, cefepime, and meropenem collected samples

In three sets of 5 mL centrifuge tubes, 1 mL of each sample was accurately transferred to which 100 μL of IS stock solution (1 mg mL^−1^) was added. The volume was then completed to 2 mL with methanol. Samples were vortexed for one min and then plasma protein was separated by centrifugation for 5 min at 4000 rpm. 20 μL of the clear supernatant from each sample was then applied in triplicates to HPTLC plates.

Chromatographic development was then done following the instructions under chromatographic conditions. The peak area ratio was then calculated and used to calculate the concentrations of each drug in the collected plasma samples using the previously computed regression equations.

## Results and discussion

Therapeutic drug monitoring of antibiotics is essential to diminish the spreading of bacterial resistance, so, it is necessary to develop reliable analytical methods for their therapeutic monitoring and quality control analysis. β-Lactam antibiotics are still known for their excellent safety and efficacy profile, they work by interfering with bacterial cell wall synthesis which is absent in human cell.^50^ From the literature survey discussed above, no HPTLC method was published for the simultaneous quantitation of different β-lactam antibiotics in real plasma samples. Thin layer chromatography is solid liquid chromatographic method at which the separation depends on the difference in solubility and adsorption of different compounds between two phases (mobile phase and stationary phase) at which they are to be portioned. The developed HPTLC method has advantages of short analysis time and low solvent consumption which are economically effective. In addition, solvents used have harmless environmental impact which is essential in the field of green chemistry.

### Method development and optimization

The main goal during optimization of the developing system was to test green solvents and exclude harmful ones such as chloroform, methylene chloride, benzene, *etc.* Different mobile phase mixtures were tested starting with acetone : methanol (9 : 1, 7 : 3, and 6 : 3, v/v) and ethylacetate : methanol (9 : 1, 7 : 3, and 6 : 3, v/v). In both cases, no complete separation among the studied drugs and plasma was observed, moreover, spots of meropenem, cefepime, and plasma appeared near the baseline. However, ethylacetate was preferred than acetone as it gave compact spots and the ratio (6 : 3, v/v) was chosen. Looking at the structure of the studied drugs, it was found that they contained both acidic and basic groups, hence their chromatographic resolution was expected to be pH dependent. Different ratios of ammonia solution (33%), triethylamine, acetic acid, and formic acid were separately tested (0.5, 1, and 1.5%). Adjusting the medium basic with either ammonia solution or triethylamine resulted in unresolved spots between cefotaxime and cefaclor. In one hand, changing the medium with acetic acid, meropenem and cefepime co-eluted with nearly the same *R*_f_ values. On the other hand, using formic acid (1%) resulted in a reasonable separation between all spots. Water in different ratios was then added (5, 10, and 15%) in a trial to improve separation between spots of plasma and cefepime. It was noticed that addition of 15% water improved the shape of all the separated spots and produced the best separation.

The use of internal standard during bio-analytical method development is important to correct for the variability in the analyte loss during sample treatment. Different internal standards were tested and the best one regarding the chromatographic behaviors and separation was valacyclovir. Sensitivity is an important factor for methods applied to biological fluids, different scanning wavelengths (230, 254, 270, and 290 nm) were tested and the highest sensitivity was obtained on scanning at 270 nm for all the proposed β-lactam antibiotics. In the same way, time of equilibration required before development is important to attain homogeneity of the atmosphere, thus diminishes the evaporation of the solvent from the HPTLC plate during the development. Effect of mobile phase saturation time on the chromatographic separation was tested (15 and 30 min.), where no considerable effect was revealed.

**Fig. 1:**
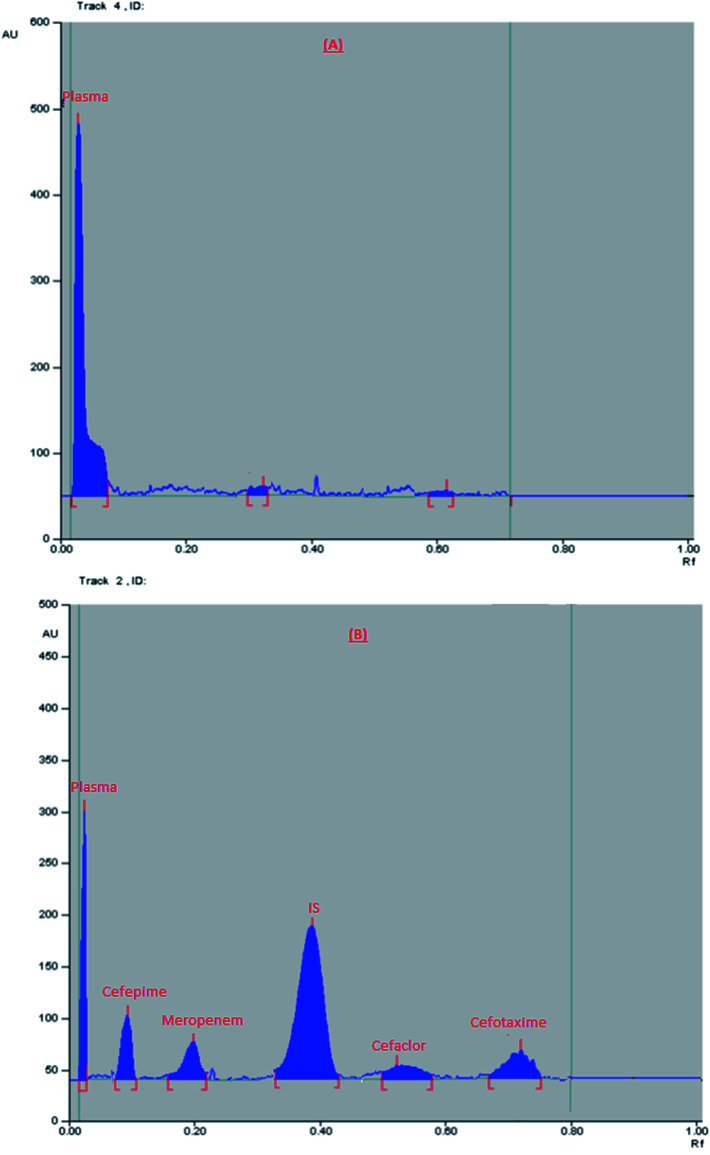
2D chromatogram of (A) blank plasma, (B) plasma sample spiked with a mixture of the studied drugs (at their LLOQ) and internal standard.

Finally, the optimum conditions were; mobile phase mixture of ethyl acetate : methanol : water : formic acid (6 : 3 : 1.5 : 0.1, by volume), the saturation time was 15 min., and UV scanning at 270 nm. The obtained *R*_f_ values were: 0.04, 0.1, 0.19, 0.38, 0.55, and 0.76 for plasma, cefepime, meropenem, IS, cefaclor, and cefotaxime, respectively, Fig. 1.

After optimization of the method, linearity was first checked by construction of calibration curves using pure standards of the studied antibiotics. Calibration curves relating the peak area ratio (area of the pure standard/area of IS) to the corresponding concentrations were plotted and linearity was proven using polynomial regression in the concentration ranges of 0.06–3 μg per band for all the proposed drugs. The computed regression equations are given in Table 1.

**Table 1:** Assay and method validation parameters for the determination of the studied drugs by the proposed method^a^

Parameters	Pure samples				Spiked human plasma samples			
	Cefaclor	Cefotaxime	Cefepime	Meropenem	Cefaclor	Cefotaxime	Cefepime	Meropenem
Range, μg per band	0.06–3.0				0.1–3.0			
Slope	−0.0661^a^	−0.1419^a^	−0.0891^a^	−0.0503^a^	−0.0328^a^	−0.0103^a^	−0.0301^a^	−0.0560^a^
	0.6292^b^	1.0245^b^	0.4649^b^	0.4006^b^	0.4271^b^	0.4765^b^	0.4218^b^	0.4423^b^
Intercept	0.0059	0.0195	0.0288	0.0151	−0.0048	0.0095	0.0468	0.0421
Correlation (*r*)	0.9996	0.9998	0.9996	0.9994	0.9996	0.9998	0.9993	0.9994
LLOQ	—				0.10			
ULOQ	—				3.00			

a The linearity was achieved using the polynomial regression equation: *A* = *aX*^2^ + *bX* + *C*. ^a^: coefficient 1. ^b^: coefficient 2. *A* = peak area ratio (peak area of the analyte/peak area of IS), *X*= concentration μg per band. *C* = intercept.

### Method validation

Validation of the proposed analytical method was carried out following FDA^45^ guidelines for bio-analytical method validation.

#### Linearity of calibration curves, lower limits of quantification, and quality control samples

Calibration range of plasma samples spiked with the studied drugs was established in the concentration range of 0.1–3 μg per band for all the proposed antibiotics using peak area ratio and polynomial regression, Table 1. Moreover, lower limit of quantitation (LLOQ) was tested and considered to be the lowest concentration on the calibration curve that have a precision with % RSD ≤ 20% and its accuracy should be within 100 ± 20% of the actual concentration. LLOQ was found to be 0.1 μg per band for cefaclor, cefotaxime, cefepime, and meropenem. Additionally, upper limit of quantitation (ULOQ) was tested to be the highest concentration on the plotted calibration curve with precision of % RSD ≤ 15% and accuracy within 100 ± 15%. ULOQ was calculated and found to be 3 μg mL^−1^ for the studied four components.

#### Quality control samples (QCs)

Three QC samples were chosen, the first one (low QC (LQC)) should be at least three times as LLOQ, the second sample was (middle QC (MQC)), which should be within the middle of the calibration curve, and the third sample was chosen to be at the high end of the calibration graph and lower than ULOQ and it was called (high QC (HQC)). The selected QC samples were 0.6, 1.5, and 2.5 μg per band for all the studied β-lactam antibiotics.

Concentrations of the samples of calibration standard and QC samples were calculated using the computed regression equations given in Table 1. The results of each sample (calibration standards and quality control samples) were accepted when deviation is not more than 15% from the true concentrations (100 ± 15%) while that of LLOQ was accepted when its deviation was ≤20% (100 ± 20%) from the nominal concentration.

#### Specificity and selectivity

**Fig. 2:**
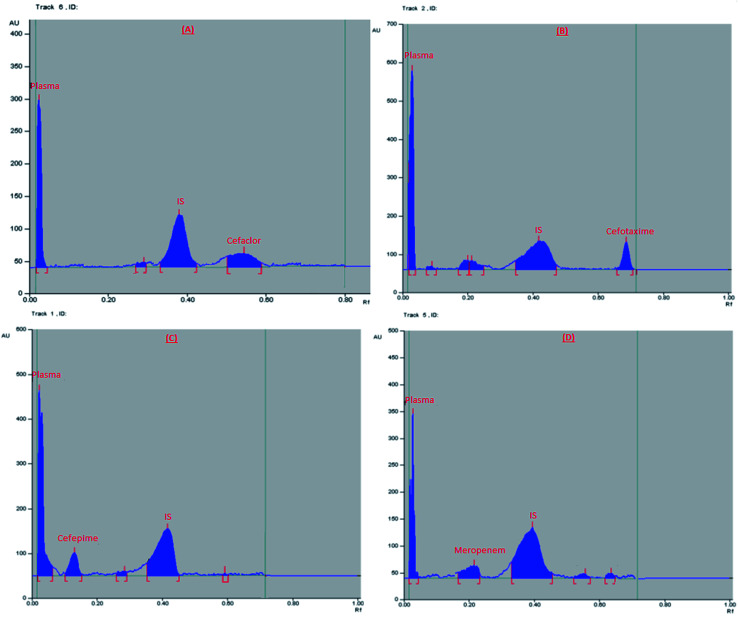
2D chromatogram of real plasma samples obtained from healthy volunteers at their *t*_max_.

Specificity of the method is the ability of the method to differentiate between the analyte of interest and the endogenous plasma components. It was tested by comparing HPTLC chromatograms obtained from applying blank plasma samples (from six different plasma batches), plasma samples spiked with each of the studied four drugs at their LLOQ and IS, and plasma samples obtained from healthy volunteers received the specified dose of each drug. HPTLC chromatograms in Fig. 1 and 2 proved that there was no interference among the studied components and plasma matrix, and confirmed selectivity of the method.

#### Accuracy and precision

Four samples (LLOQ and QC samples) were used to evaluate accuracy and precision of the developed HPTLC method. Within run accuracy and precision was tested by analysis of the four samples (*n* = 3) in the same day while between run precision and accuracy was checked by analysis of the prepared samples (*n* = 3) on three successive days. The resulted concentrations were calculated from the previously computed regression equations. Accuracy was expressed as the bias [[(calculated concentration − actual concentration)/actual concentration] × 100], while precision was expressed as the coefficient of variation (CV) or % RSD [(SD/mean) × 100]. Results in Table 2 were within the acceptance limits (bias was within ±15% for QC samples and ±20% for LLOQ while CV was ≤15% for QC samples and ≤20% for LLOQ).

**Table 2:** Intra and inter assay precision and accuracy

Component	Concentration^a^ (μg per band)	Intraday			Interday		
		Recovery%	RSD%	Bias%^b^	Recovery%	RSD%	Bias%^b^
Cefaclor	0.10 (LLOQ)	102.56	7.29	2.56	91.98	8.13	−8.02
	0.60 (LQC)	108.02	2.83	8.02	99.79	8.60	−0.21
	1.50 (MQC)	99.57	0.04	−0.43	109.34	9.40	9.34
	2.50 (HQC)	104.64	7.49	4.64	104.86	9.72	4.86
Cefotaxime	0.10 (LLOQ)	105.5	8.47	5.75	93.07	9.63	−6.93
	0.60 (LQC)	93.97	5.09	−6.03	107.56	13.42	7.56
	1.50 (MQC)	94.32	7.92	−5.68	91.48	10.09	−8.52
	2.50 (HQC)	99.13	6.76	−0.87	89.66	7.47	−10.30
Cefepime	0.10 (LLOQ)	116.77	3.91	16.77	118.46	1.84	18.46
	0.60 (LQC)	105.52	4.42	5.52	103.65	6.24	3.65
	1.50 (MQC)	106.34	5.77	6.34	108.67	9.55	3.65
	2.50 (HQC)	96.21	6.53	−3.79	95.29	9.15	−4.71
Meropenem	0.10 (LLOQ)	102.56	4.57	2.56	107.22	10.53	7.22
	0.60 (LQC)	98.74	6.19	−1.26	109.01	3.31	8.99
	1.50 (MQC)	90.00	4.20	−10.00	97.67	9.17	−2.33
	2.50 (HQC)	106.35	0.31	6.35	99.21	8.84	−0.79

a Average of 3 experiments.

b Bias = [(measured concentration − nominal concentration)/nominal concentration] × 100.

#### Extraction recovery

Extraction recovery was calculated for both the studied analytes and IS and it was evaluated by comparing the peak areas for the extracted QC samples with those of un-extracted standards (represent 100% recovery). Results should be reproducible to be acceptable, regardless the resulting extraction recovery. The mean extraction recoveries were calculated and found to be 92.46 ± 3.862, 76.75 ± 4.946, 91.71 ± 5.459, and 90.01 ± 1.316 for cefaclor, cefotaxime, cefepime, and meropenem, respectively. The results given in Table 3 confirmed the efficiency of the extraction procedure followed in this work.

**Table 3:** Extraction recovery results of the studied drugs in spiked human plasma

Analyte	Concentration of the analyte (μg per band)	% recovery^a^
Cefaclor	0.60	88.56
	1.50	93.25
	2.50	95.57
Mean ± % RSD		92.46 ± 3.862

Cefotaxime	0.60	76.86
	1.50	73.19
	2.50	80.80
Mean ± % RSD		76.75 ± 4.946

Cefepime	0.60	91.24
	1.50	86.96
	2.50	96.94
Mean ± % RSD		91.71 ± 5.459

Meropenem	0.60	94.95
	1.50	95.68
	2.50	97.41
Mean ± % RSD		90.01 ± 1.316

IS	1.00	95.07 ± 2.987

a Average of 3 determinations.

#### Stability of quality control samples

The stability was tested by exposing QC samples to variable stability conditions such as bench-top stability and freeze–thaw stability. QC samples were stored at room temperature (25 °C) for 6 h to test bench-top stability and also were subjected to three freeze–thaw cycles (from freezing at −20 °C for 12 h to room temperature (freeze and thaw stability)). Sample was considered stable when the change in the analyte concentration was ≤15%. Results presented in Table 4 assured that samples concentrations did not significantly changed under the tested stability conditions.

**Table 4:** Stability results of the studied drugs in spiked human plasma at different conditions

Analyte	% recovery^a^		
	Concentration of the analyte (μg per band)	Three freeze–thaw cycles	Bench top stability
Cefaclor	0.60	103.79	96.72
	1.50	99.30	111.39
	2.50	89.90	109.67
Mean ± % RSD		97.66 ± 7.256	105.93 ± 7.57

Cefotaxime	0.60	106.89	99.63
	1.50	97.54	107.50
	2.50	98.78	96.8
Mean ± % RSD		101.07 ± 5.024	101.31 ± 5.473

Cefepime	0.60	108.37	89.87
	1.50	107.18	92.37
	2.50	110.63	89.78
Mean ± % RSD		108.73 ± 1.612	90.67 ± 1.621

Meropenem	0.60	99.08	110.13
	1.50	96.24	111.33
	2.50	104.79	100.00
Mean ± % RSD		100.04 ± 4.353	107.15 ± 5.808

a Average of 3 determinations.

### Results of method application to real human plasma samples

The developed HPTLC method was successfully used to determine the concentration of a single dose of the studied antibiotics in real human plasma of healthy volunteers. From previous studies, it was reported that *C*_max_ (μg mL^−1^) for the given doses was 18.16, 102, 78.7, and 70 μg mL^−1^ for cefaclor, cefotaxime, cefepime, and meropenem, respectively.^46–49^ Chromatograms obtained from selected plasma samples are shown in Fig. 2. Additionally, the concentrations of the given doses were calculated from the previously computed regression equations and summarized in Table 5. All the plasma concentrations measured in volunteers samples were within the calibration range of the proposed HPTLC method.

Results of determination of the studied drugs in real human plasma

**Table:** 

Volunteers	Expected concentration (μg per band)^46^	The found concentration (μg per band)	Expected concentration (μg per band)	The found concentration (μg per band)^47^
	Cefaclor		Cefotaxime	
1	0.454	0.480	1.020	1.120
2		0.476		0.800
3		0.570		1.187
4		0.510		1.053
5		0.560		1.144
6		0.420		1.013
Mean ± % RSD		0.519 ± 10.873		1.053 ± 13.184

**Table:** 

Patients	Expected concentration (μg per band)^48^	The found concentration (μg per band)	Expected concentration (μg per band)^49^	The found concentration (μg per band)
	Cefepime		Meropenem	
1	0.787	0.890	0.700	0.575
2		0.720		0.712
3		0.707		0.637
4		0.827		0.688
5		0.613		0.588
6		0.693		0.737
Mean ± % RSD		0.742 ± 13.467		0.656 ± 10.170

## Conclusion

An innovative HPTLC method was established for *in vivo* analysis of four β-lactam antibiotics. The method had the advantages of simple sample preparation and short analysis time. Moreover, the proposed method adopted the use of green solvents with harmless environmental impact. All validation parameters agreed with FDA acceptance criteria. This method permitted the accurate determination of the studied antibiotics thus, it can be used during therapeutic drug monitoring in daily clinical practice, hence minimize the antibiotics microbial resistance.

## Ethics

The study was approved by the Animal Care and Use Committee of the Faculty of Medicine, Beni-Suef University (FM-BSU REC) according to guidelines of the declaration of Helsinki, International Conference on Harmonization (ICH) and United States Codes of Federal Regulation and registered in under the Federal Wide Assurance number (REC-A-PHBSU18005) for protection of animals (appendix 1).

## Conflicts of interest

Both authors declare that they have no conflicts of interest.

## Supplementary Material
